# A placebo-controlled, double-blind, dose-escalation study to assess the safety, tolerability and pharmacokinetics/pharmacodynamics of single and multiple intravenous infusions of AZD9773 in patients with severe sepsis and septic shock

**DOI:** 10.1186/cc11203

**Published:** 2012-02-17

**Authors:** Peter E Morris, Brian Zeno, Andrew C Bernard, Xiangning Huang, Shampa Das, Timi Edeki, Steven G Simonson, Gordon R Bernard

**Affiliations:** 1 Wake Forest University School of Medicine, Winston Salem, NC, USA; 2 Riverside Methodist Hospital, Columbus, OH, USA; 3 University of Kentucky, Lexington, KY, USA; 4 AstraZeneca, Alderley Park, Macclesfield, UK; 5 AstraZeneca, Wilmington, DE, USA; 6 Agennix Incorporated, Princeton, NJ, USA; 7 Vanderbilt University, Nashville, TN, USA

## Abstract

**Introduction:**

Tumor necrosis factor-alpha (TNF-α), an early mediator in the systemic inflammatory response to infection, is a potential therapeutic target in sepsis. The primary objective of this study was to determine the safety and tolerability of AZD9773, an ovine, polyclonal, anti-human TNF-α Fab preparation, in patients with severe sepsis. Secondary outcomes related to pharmacokinetic (PK) and pharmacodynamic (PD) parameters.

**Methods:**

In this double-blind, placebo-controlled, multicenter Phase IIa study, patients were sequentially enrolled into five escalating-dose cohorts (single doses of 50 or 250 units/kg; multiple doses of 250 units/kg loading and 50 units/kg maintenance, 500 units/kg loading and 100 units/kg maintenance, or 750 units/kg loading and 250 units/kg maintenance). In each cohort, patients were randomized 2:1 to receive AZD9773 or placebo.

**Results:**

Seventy patients received AZD9773 (*n *= 47) or placebo (*n *= 23). Baseline characteristics were similar across cohorts. Mean baseline APACHE score was 25.9. PK data demonstrated an approximately proportional increase in concentration with increasing dose and a terminal half-life of 20 hours. For the multiple-dose cohorts, serum TNF-α concentrations decreased to near-undetectable levels within two hours of commencing AZD9773 infusion. This suppression was maintained in most patients for the duration of treatment. AZD9773 was well tolerated. Most adverse events were of mild-to-moderate intensity and considered by the reporting investigator as unrelated to study treatment.

**Conclusions:**

The safety, PK and PD data support the continued evaluation of AZD9773 in larger Phase IIb/III studies.

## Introduction

Sepsis remains a major cause of mortality, despite significant advances in antibiotic therapy and medical technology [1,2]. Current options for managing sepsis include treatment of underlying infection, restoration of tissue perfusion and oxygenation, and other organ-supportive methods [3]. In addition to the symptomatic management of sepsis, modulation of the host response to the infection is a desired goal. Drotrecogin alfa (activated) recombinant human (rh) activated protein C (Xigris^®^) was, until recently, the only immunomodulatory drug specifically approved for the treatment of severe sepsis. However, the drug was withdrawn in October 2011 following the results of the placebo-controlled PROWESS-SHOCK study, where the primary endpoint of a statistically significant reduction in 28-day all-cause mortality was not met.

Based on current understanding of the inflammatory cascade, the release of cytokines into the circulation is recognized as an early and essential part of sepsis pathology. Experimental and clinical data have shown that the pro-inflammatory cytokine tumor necrosis factor-α (TNF-α) is a principal initiator of this cascade [4,5]. TNF-α is one of the first cytokines to be released by macrophages in response to infection [6] and, once in the circulation, it causes systemic inflammation through stimulating the widespread release of downstream cytokines, such as interleukin-6 (IL-6) and IL-8 [7]. Given its role as an early mediator of the inflammatory response, TNF-α is an appropriate target for the treatment of sepsis.

A large number of immunomodulatory agents have been studied in the clinical setting. However, trials with a variety of intact antibodies, fragment antigen-binding (Fab) dimers and soluble receptors against TNF have so far shown only limited signals of efficacy in sepsis [8,9]. AZD9773 is a preparation of polyclonal Fab fragments obtained from sheep immunized with rhTNF-α. AZD9773 has a number of potential advantages over previously tested agents designed to neutralize TNF-α [9]. Being a polyclonal product, it binds to more than one domain of TNF-α [10], and as a monomeric Fab fragment rather than an intact antibody or Fab'2 dimer, it is likely to exhibit improved tissue penetration [11,12]. These fragments have been shown to neutralize TNF in the lung in severe sepsis patients [13]. Antibody fragments may also have a shorter serum half-life than intact antibodies, enabling more controlled time-limited TNF suppression.

CytoFab, an earlier development formulation of AZD9773 with similar polyclonal anti-TNF-α activity, has shown the potential therapeutic benefit of polyclonal Fab fragments. In a Phase II study in patients with severe sepsis, CytoFab reduced plasma TNF-α and IL-6 levels and increased the number of ventilator- and intensive care unit (ICU)-free days compared with placebo [13]. In addition, all-cause 28-day mortality rates were higher in the placebo than the CytoFab group (37 vs 26%; *P *= 0.274). Since completion of this study, significant manufacturing changes have been introduced to ensure quality and to facilitate increased production for more extensive study of the drug product. The key modification relates to the chromatographic purification of the ovine Fab fragments, where an affinity chromatography step has been replaced with sequential anion and cation exchange steps. The resultant product (AZD9773) contains a substantially higher concentration of non-specific Fab fragments than CytoFab. *In vitro *cell-based assays [14] and *in vivo *primate pharmacodynamic studies [15] confirm that the increased fraction of non-TNF-α-directed Fabs does not negatively affect the potency of the drug product. The only apparent pharmacological difference is that AZD9773 has a lower biological activity (measured as TNF-α neutralizing units) per mg protein [15]; therefore, in order to deliver an equivalent unit dose, the protein dose of AZD9773 is higher than that required for CytoFab.

The objectives of the current study were to characterize the safety and tolerability of various single and multiple doses of AZD9773 in patients with severe sepsis, and also to perform a preliminary assessment of the pharmacokinetics (PK) and pharmacodynamics (PD) of AZD9773.

## Materials and methods

### Study design

This was a first-in-man, multicenter, double-blind, dose-escalation Phase IIa study (clinicaltrials.gov identifier: NCT00615017) to assess single and multiple (a loading dose followed by maintenance doses given every 12 hours for five days) intravenous infusions of AZD9773 versus placebo (2:1) in patients with severe sepsis. The study comprised a treatment period (Day 1 for single-dose cohorts, Days 1 to 6 for multiple-dose cohorts), then a follow-up period to Day 28. The institutional review board and an independent ethics committee at each site approved the study protocol and informed consent was obtained from each subject or their legal representative. The study was conducted in accordance with Good Clinical Practice guidelines and the Declaration of Helsinki.

### Patient population

Key inclusion criteria included: male or female patients aged ≥ 18 years with severe sepsis; clinical evidence of infection; systemic inflammatory response syndrome (SIRS); cardiovascular and/or respiratory organ dysfunction (according to the study definitions - see Additional files 1, 2, 3). Study drug had to be administered within 36 hours of the qualifying organ dysfunction, which itself had to occur within 24 hours of the SIRS criterion. Before receiving the first dose of study drug, each patient was required to have received at least one dose of parenteral antibiotics. Key exclusion criteria included: human immunodeficiency virus infection with a last known CD4 count ≤ 50/mm^3^; patient moribund, with death considered imminent (within 24 hours of sepsis recognition); patient not expected to survive 90 days because of an underlying medical condition; organ or bone marrow transplant within the previous 6 months; immunosuppressant therapy or treatment with anti-TNF antibodies within the previous 2 months; active tuberculosis or severe chronic respiratory disease. The use of immunosuppressant drugs, high-dose steroids and anti-TNF antibodies other than the study drug was prohibited during the study, as was administration of the study drug outside of the ICU. Drotrecogin alfa (activated) use was allowed at the discretion of the investigator.

### Dosing

Patients were sequentially recruited into five cohorts and then randomized to receive AZD9773 (Table 1) or placebo; AZD9773 was administered as an intravenous infusion.

**Table 1:** AZD9773 dosing regimens

	AZD9773 cohort 1	AZD9773 cohort 2	AZD9773 cohort 3	AZD9773 cohort 4	AZD9773 cohort 5
Loading dose × 1	**50 units/kg** (over 90 minutes)	**250 units/kg** (over 225 minutes for first three patients, then 95 minutes for next nine patients)	**250 units/kg** (over 65 minutes for first three patients, then 60 minutes for next nine patients)	**500 units/kg** (over 90 minutes for first three patients, then 60 minutes for next nine patients)	**750 units/kg** (over 75 minutes for first three patients, then 60 minutes for next nine patients)
Maintenance doses × 9			**50 units/kg** (over 30 minutes once every 12 hours)	**100 units/kg** (over 30 minutes once every 12 hours)	**250 units/kg** (over 30 minutes once every 12 hours)

Prior to use, AZD9773 was reconstituted with saline. Maximum dose infusion volumes were either 100 or 250 mL (cohorts 4 or 5 loading dose only). Patients with a body weight > 100 kg received doses corresponding to 100 kg. Placebo (0.9% saline solution) was administered as an intravenous infusion in an equivalent volume to the active treatment; blinding was maintained using masking bags sealed with tamper-evident tape.

### Assessments

#### Pharmacokinetic and pharmacodynamic assessments

Blood and urine samples were collected daily for PK assessments of both AZD9773-specific and total Fabs (TNF-α-specific Fab and all other non-TNF-directed Fabs present in AZD9773). AZD9773-specific and total Fabs were quantitatively determined using a direct sandwich ELISA format (Quotient Bioresearch, Fordham, Cambridgeshire, UK) with specific detection reagents to the total ovine IgG Fab fragments or against the anti-TNF-α binding moiety of the drug. Concentrations were determined using AZD9773 standard curves. Blood samples for cohorts 1 and 2 were collected pre-infusion, at the end of the infusion and at 0.5, 1, 2, 4, 8, 12, 24, 48 and 72 hours post-infusion, while those for cohorts 3 to 5 were collected pre-infusion for the loading dose and maintenance doses 5, 7 and 9 (that is, the last one), and at the end of the infusion and at 0.5, 1, 2, 4, 8 and 12 hours post-infusion for the loading dose and last maintenance dose. Urine samples for cohorts 1 and 2 were collected pre-infusion and for 72 hours post-infusion, while those for cohorts 3 to 5 were collected pre-infusion and for 12 hours post-infusion for the loading and last maintenance dose.

PD assessments included measurement of TNF-α and IL-6 in serum using the ELISA-based Quantikine high-sensitivity human TNF-α/TNFSF1A enzyme immunoassay (R&D Systems, Minneapolis, MN, USA). In this system a monoclonal antibody is used to capture TNF-α present in the patient sample; bound TNF-α is then detected using an enzyme-linked polyclonal antibody specific for TNF-α. Blood samples were drawn pre-infusion, 2 and 3 hours after the initial infusion ended (for TNF-α only), and 24, 48, 72, 96, 120, 144 and 168 hours after start of infusion. The limit of quantification (LoQ) was 1.3 pg/mL for TNF-α [16] and < 0.70 pg/mL for IL-6 [17]. Procalcitonin serum concentration, as an exploratory marker of infection, was assessed at baseline and Days 1, 5 and 7, using the LUMItest PCT immunoluminometric assay (Brahms Diagnostic, Berlin, Germany); normal procalcitonin levels are < 0.5 ng/mL [18]. All sample analyses were conducted by Covance Central Laboratory Services, Geneva, Switzerland.

#### Safety

Safety was assessed based on the frequency and severity of treatment-emergent adverse events (TEAEs; defined as events not present at baseline or that worsened in severity following the start of treatment), serious TEAEs, assessment of laboratory parameters, electrocardiogram (ECG) measurements, and all-cause 28-day mortality. AEs were assessed regularly throughout the study, were coded according to the Medical Dictionary for Regulatory Activities (MedDRA v12.0) and were assumed to be treatment emergent unless there was clear evidence that the event was present at the first dose of study drug. Blood samples for the laboratory assessment of hematology, clinical chemistry and urinalysis parameters were collected daily during treatment, as well as after 14 and 28 days. ECG measurements were performed daily before infusion, at the end of infusion and approximately 4, 6 and 12 hours post-infusion (also 24 hours post-infusion for AZD9773 cohorts 1 and 2).

#### General sepsis outcomes

Sepsis outcomes were evaluated based on:

■ Sequential Organ Failure Assessment (SOFA) score [19]: comprised six individual component scores. Failure of an organ system was recorded the first time any of the criteria specified in the individual components of the SOFA score were > 1 after the start of the study drug up to Day 14. Organ failure-free days were days when the organ system SOFA score was ≤ 1; these were assessed up to Day 14.

■ Organ failure assessment: measured using SOFA scores collected up to Day 14. Total organ failure resolution was defined as resolution of all organ failures (either those present at baseline or starting post-baseline).

■ Ventilator use: the number of ventilator-free days was measured and defined as the number of days after starting unassisted breathing to Day 28, assuming a patient survived for at least two consecutive calendar days after starting unassisted breathing and remained free of assisted breathing.

■ Treatment-emergent infections: the number of patients with positive cultures occurring prior to, or infections (reported as AEs) occurring after, first dose was assessed.

■ Quality of background care was characterized and evaluated, in part, by assessment of time to first dose of antibiotics, adequacy of fluid resuscitation, tidal volume in patients receiving mechanical ventilation, and the proportion of patients receiving stress ulcer and deep vein thrombosis prophylaxis.

#### Human anti-sheep antibody (HASA) IgG and bridging assay

Anti-AZD9773 antibodies were semi-quantitatively determined in human serum by utilizing ELISA antibody screening assays [20-22]. Ninety-six-well microtitre plates were coated with AZD9773 and incubated with the unknown sample or positive controls. The anti-AZD9773 antibodies present in the sample, or positive control anti-serum from rabbit immune serum added to the wells, bound to AZD9773 immobilized on the microtitre plate. Detection was via addition of biotinylated AZD9773 and ExtrAvidin-HRP. A cut-point factor was applied to each plate for data interpretation. Confirmatory and titration assays were carried out using the same format. Samples were taken at baseline, Day 7 and Day 28.

### Statistical analysis

The determination of sample size was based on practical requirements rather than any formal statistical sample size calculation. The number of patients was selected to facilitate collection of sufficient safety and PK/PD data to inform the design of future sepsis studies with AZD9773, without unnecessarily exposing patients to the drug. Descriptive statistics were applied to summarize the continuous data, including number of observations, mean, geometric mean, standard deviation (SD), coefficient of variation (%CV), median, minimum and maximum. Categorical data were summarized in terms of frequency counts and percentages. A significance level of *P <*0.05 was employed in exploratory analyses.

The safety population comprised all patients who started an infusion of the study drug. The PK population was a subset of the safety population, excluding patients who had dosing errors that could affect the PK of AZD9773.

### Data and safety monitoring board

A Safety Review Committee reviewed safety data at regular intervals during the study and assessed the planned dose escalation schedule. Additionally, an Independent Data Monitoring Committee (IDMC) met at planned intervals to review study issues and unblinded safety data. IDMC procedures were described in the IDMC Charter and were agreed to by each committee member.

## Results

### Patient disposition

**Figure 1:**
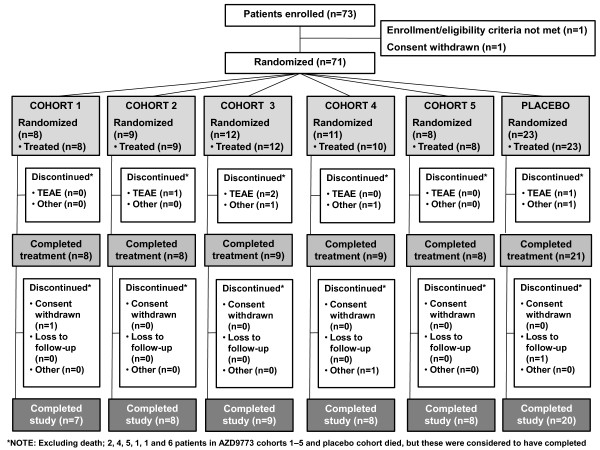
**Patient disposition**.

The study was conducted at 27 centers in the USA from January 2008 until July 2009. Of 73 patients enrolled, 71 were randomized and 70 received treatment (*n *= 47, AZD9773; *n *= 23, placebo). One patient in AZD9773 cohort 4 died before receiving the drug and was excluded from the safety and PK analyses. Three other patients were excluded from the PK analysis due to dosing errors. Overall (and classifying patients who died as completers), 63 patients (90.0%) completed the treatment period and 60 (85.7%) completed the 28-day study period (Figure 1).

### Baseline demographics and disease characteristics

The baseline demographic and disease characteristics were generally balanced across the treatment groups and were typical of patients with severe sepsis and/or septic shock (Table 2).

**Table 2:** Baseline demographic and disease characteristics (safety population)

	AZD9773 cohort 1 (50 U/kg) (*n *= 8)	AZD9773 cohort 2 (250 U/kg) (*n *= 9)	AZD9773 cohort 3 (250/50 U/kg) (*n *= 12)	AZD9773 cohort 4 (500/100 U/kg) (*n *= 10)	AZD9773 cohort 5 (750/250 U/kg) (*n *= 8)	Placebo (*n *= 23)	Total (*n *= 70)
Mean age ± SD, years	53.0 ± 11.8	49.7 ± 15.0	55.1 ± 10.8	53.0 ± 17.9	63.5 ± 16.1	58.9 ± 17.4	56.1 ± 15.5
Male:female, n	6:2	5:4	5:7	3:7	2:6	11:12	32:38
Race, n (%)							
Caucasian/white	7 (87.5)	8 (88.9)	11 (91.7)	9 (90.0)	7 (87.5)	20 (87.0)	62 (88.6)
Black/African American	1 (12.5)	1 (11.1)	1 (8.3)	0	1 (12.5)	2 (8.7)	6 (8.6)
Other	0	0	0	1 (10.0)	0	1 (4.3)	2 (2.9)
Shock, n (%)							
Yes	5 (62.5)	6 (66.7)	9 (75.0)	9 (90.0)	7 (87.5)	20 (87.0)	56 (80.0)
No	3 (37.5)	3 (33.3)	3 (25.0)	1 (10.0)	1 (12.5)	3 (13.0)	14 (20.0)
On ventilator, n (%)*							
Yes	7 (87.5)	8 (100.0)	9 (81.8)	8 (88.9)	8 (100.0)	20 (87.0)	60 (89.6)
No	1 (12.5)	0	2 (18.2)	1 (11.1)	0	3 (13.0)	7 (10.4)
Missing	0	1	1	1	0	0	3
Shock + ventilator, n (%)*	4 (50.0)	5 (55.6)	7 (58.3)	7 (70.0)	7 (87.5)	17 (73.9)	47 (67.1)
Organ failures, n (%)**							
0	1 (12.5)	0	1 (8.3)	0	0	0	2 (2.9)
1	0	0	2 (16.7)	2 (20.0)	2 (25.0)	1 (4.3)	7 (10.0)
2	2 (25.0)	3 (33.3)	2 (16.7)	1 (10.0)	1 (12.5)	5 (21.7)	14 (20.0)
3	3 (37.5)	4 (44.4)	3 (25.0)	3 (30.0)	1 (12.5)	9 (39.1)	23 (32.9)
≥ 4	2 (25.0)	2 (22.2)	4 (33.3)	4 (40.0)	4 (50.0)	8 (34.8)	24 (34.3)
APACHE II score	*(n = 8)*	*(n = 9)*	*(n = 12)*	*(n = 10)*	*(n = 7)*	*(n = 22)*	*(n = 68)*
Mean ± SD	28.4 ± 10.3	25.1 ± 7.8	26.3 ± 11.1	22.9 ± 9.0	25.1 ± 8.8	26.6 ± 8.0	25.9 ± 8.9
SOFA score	*(n = 7)*	*(n = 8)*	*(n = 7)*	*(n = 8)*	*(n = 6)*	*(n = 20)*	*(n = 56)*
Mean ± SD	11.9 ± 4.3	10.9 ± 3.6	10.4 ± 3.4	11.5 ± 2.6	10.3 ± 3.6	10.7 ± 3.0	11.0 ± 3.2
TNF-α concentration, pg/mL	*(n = 8)*	*(n = 8)*	*(n = 9)*	*(n = 9)*	*(n = 8)*	*(n = 23)*	*(n = 65)*
Median (range)	4.7 (1.3 to 48.8)	9.8 (2.7 to 54.3)	3.6 (1.3 to 14.2)	3.7 (1.3 to 8.2)	5.8 (1.3 to 18.3)	5.0 (1.6 to 61.7)	5.2 (1.3 to 61.7)
IL-6 concentration, pg/mL	*(n = 7)*	*(n = 7)*	*(n = 9)*	*(n = 8)*	*(n = 7)*	*(n = 19)*	*(n = 57)*
Median (range)	465 (18 to 23,733)	189 (47 to 85,183)	215 (24 to 13,849)	1403 (135 to 16,168)	336 (194 to 94,207)	454 (28 to 153,600)	336 (18 to 153,600)
Procalcitonin level, ng/mL	*(n = 8)*	*(n = 8)*	*(n = 10)*	*(n = 10)*	*(n = 6)*	*(n = 23)*	*(n = 65)*
Median (range)	28.5 (1.8 to 456.4)	4.8 (0.3 to 250.9)	4.0 (0.3 to 800.3)	16.5 (1.3 to 89.6)	15.1 (2.5 to 17.1)	14.7 (0.3 to 328.5)	14.2 (0.3 to 800.3)
> 0.5 ng/mL, n (%)	8 (100)	7 (87.5)	7 (70.0)	10 (100)	6 (100)	22 (95.7)	60 (92.3)
Total fluid volume in 24 h prior to randomization, mL/kg	*(n = 8)*	*(n = 9)*	*(n = 11)*	*(n = 7)*	*(n = 8)*	*(n = 23)*	*(n = 66)*
Median (range)	51.7 (23.8 to 167.6)	58.0 (6.4 to 158.6)	40.1 (11.1 to 183.4)	34.0 (17.2 to 196.8)	58.5 (29.3 to 434.1)	58.4 (8.7 to 373.5)	56.4 (6.4 to 434.1)
Relevant medical history, n (%)^†^							
Diabetes	1 (12.5)	4 (44.4)	4 (33.3)	3 (30.0)	3 (37.5)	7 (30.4)	22 (31.4)
COPD	3 (37.5)	2 (22.2)	2 (16.7)	2 (20.0)	3 (37.5)	4 (17.4)	16 (22.9)
Chronic renal failure	1 (12.5)	2 (22.2)	0	1 (10.0)	0	0	4 (5.7)
Colon cancer	0	0	0	0	1 (12.5)	1 (4.3)	2 (2.9)

*Percentages exclude patients with a missing assessment; **Number of organ failures (SOFA score components > 1); ^†^Reported in > 1 patient overall; COPD, chronic obstructive pulmonary disease. Note: One patient in AZD9773 cohort 1 was recorded as having no organ failure as study drug was administered outside the protocol-defined time limit. However, this resulted from a data entry error that was not amended in the clinical database; the patient did in fact receive study drug within the defined time limit. One patient in AZD9773 cohort 3 was recorded as having no organ failure as they received study drug outside the protocol-defined time limit; inclusion of this patient was a protocol deviation

Twenty-six patients (55.3%) across AZD9773 cohorts had a culture-positive infection prior to study entry, compared to eight patients (34.8%) in the placebo group. The most commonly reported sites of infection at baseline were the lung and abdomen (Table 3). The overall median time from qualifying organ failure to start of treatment was 21.5 hours (range 3 to 32 hours). Across the study, antibiotics were initiated a median of 4.2 hours before first qualifying organ failure.

**Table 3:** Sites of clinically suspected infections and use of antibiotics at baseline (safety population)

	AZD9773 cohort 1 (50 U/kg) (*n *= 8)	AZD9773 cohort 2 (250 U/kg) (*n *= 9)	AZD9773 cohort 3 (250/50 U/kg) (*n *= 12)	AZD9773 cohort 4 (500/100 U/kg) (*n *= 10)	AZD9773 cohort 5 (750/250 U/kg) (*n *= 8)	Placebo (*n *= 23)	Total (*n *= 70)
Infection site, n (%)							
Abdomen	2 (25.0)	3 (33.3)	2 (16.7)	1 (10.0)	5 (62.5)	3 (13.0)	16 (22.9)
Catheter/device	1 (12.5)	0	0	0	0	0	1 (1.4)
Lung	3 (37.5)	4 (44.4)	6 (50.0)	6 (60.0)	2 (25.0)	12 (52.2)	33 (47.1)
Skin and soft tissue	1 (12.5)	1 (11.1)	1 (8.3)	1 (10.0)	0	4 (17.4)	8 (11.4)
Unknown	1 (12.5)	1 (11.1)	2 (16.7)	2 (20.0)	0	1 (4.3)	7 (10.0)
Urinary tract	0	0	3 (25.0)	0	1 (12.5)	3 (13.0)	7 (10.0)

Time from qualifying organ failure to first antibiotic use, hours*							
	*(n = 7)*	*(n = 6)*	*(n = 9)*	*(n = 7)*	*(n = 8)*	*(n = 19)*	*(n = 56)*
Median (range)	-4.4 (-39.0 to 20.6)	-10.1 (-85.7 to 0.7)	-0.8 (-41.3 to 16.4)	-3.9 (-108.2 to 1.2)	-6.3 (-85.1 to 6.5)	-4.9 (-366.5 to 12.8)	-4.2 (-366.5 to 20.6)

*A negative time indicates that antibiotics were given before qualifying organ failure

### Pharmacokinetics of AZD9773

Maximum serum concentrations of AZD9773 total Fabs were achieved at the end of infusion (EOI); levels subsequently decreased in a multi-phasic manner. There was an initial rapid decline, with concentrations decreasing by 80 to 90% within the 12 hours after EOI. Following this initial decline, a terminal phase of approximately 18 to 20 hours was observed for both single-dose cohorts. The volume of distribution and clearance was determined from the single-dose cohorts and remained comparable across doses (Table 4).

**Table 4:** Single- and multiple-dose PK of AZD9773 total Fabs

	AZD9773 cohort 1 (50 U/kg) (*n *= 7)	AZD9773 cohort 2 (250 U/kg) (*n *= 8)	AZD9773 cohort 3 **(250/50 U/kg) (*n *= 6)**^§^	AZD9773 cohort 4 **(500/100 U/kg) (*n *= 7)**^§^	AZD9773 cohort 5 (750/250 U/kg) **(*n *= 4)**^§^
AUC_0-12_, μg.h/mL*^‡^	125.2 ± 79.8	581.0 ± 94.04	449.2 ± 46.8	691.5 ± 22.7	1872 ± 28.4
AUC_0-12_, μg.h/mL*	-	-	162.8 ± 82.2	251.6 ± 56.0	981.0 ± 39.3
C_inf_, μg/mL*^‡^	29.6 ± 112.5	156.2 ± 208.1	-	-	-
C_inf_, μg/mL*	-	-	28.4 ± 52.9	42.5 ± 83.5	151.8 ± 14.7
Accumulation ratio*	-	-	1.9 ± 32.0	1.6 ± 79.1	1.6 ± 38.1
t_max_, hours**	1.5 (1.4 to 3.5)	2.1 (1.0 to 4.3)	0.5 (0 to 0.7)	1.0 (0.4 to 8.5)	0.7 (0.5 to 1.6)
t_1/2_, hours^†^	18.0 ± 7.3	19.8 ± 5.2	-	-	-
CL, mL/min/kg^†^	6.1 ± 3.4	7.6 ± 6.0	-	-	-
V_ss_, mL/kg^†^	92.6 ± 30.8	152.8 ± 132.8	-	-	-
Dose excreted, %*	10.6 ± 69.3	15.0 ± 100.4	15.2 ± 57.1	11.8 ± 94.0	20.9 ± 223.1

Data presented as: *Geometric mean ± coefficient of variation; **Median (range); ^†^Mean ± SD; ^‡^Values for single doses (cohorts 1 and 2) or loading doses (cohorts 3 to 5); ^§^Values based on maintenance doses unless stated otherwise. Note: The multiple dosing profile was not followed for sufficient time to enable the calculation of t_1/2_, CL and V_ss _for cohorts 3 to 5. AUC_0-12_, area under the serum concentration-time curve from 0 to 12 hours; C_inf_, maximum (end of infusion) serum concentration; CL, total apparent drug clearance; t_1/2_, terminal half-life; t_max_, time to reach C_inf_; V_ss_, apparent volume of distribution at steady state

Comparison of AUC_0-12 _following both single doses and loading doses from the multiple-dose cohorts indicated that exposure increased with increasing dose. A similar observation was made when comparing maintenance dose AUC_0-12 _from the multiple-dose cohorts. This increase in exposure was approximately dose proportional (Table 4). Dose normalization of the loading and maintenance doses indicated accumulation of AZD9773 following twice-daily dosing for 10 doses, with an accumulation ratio between 1.6 and 1.9. This accumulation ratio was as expected for the terminal half-life (t_1/2_) of this compound and demonstrates an absence of time dependency in the PK of AZD9773. The C_min _data confirmed that steady state was achieved by the fifth maintenance dose and were also consistent with t_1/2_.

The areas under the curves (AUCs) for specific Fabs ranged from 7.3% to 9.1% (overall mean 8.4%) of the AUC for total Fabs. This is a good agreement with the specific Fab content (8.4%) of the administered drug product. The apparent volume of distribution and t_1/2 _were similar for total and specific Fabs in the two single-dose cohorts.

There was no evidence of reduced exposure to AZD9773 in patients who received dialysis (*n *= 9). The overall proportion of dose excreted in urine across AZD9773 cohorts was approximately 15% (Table 4).

### Pharmacodynamics of AZD9773

#### TNF-α concentrations

**Figure 2:**
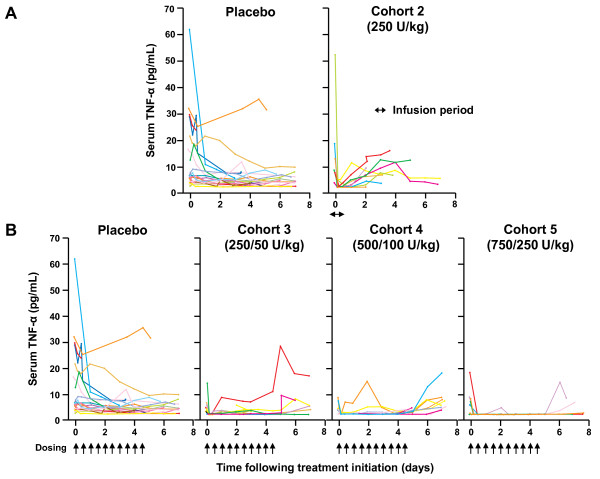
**TNF-α serum concentrations following **(A) **single 250 U/kg dose of AZD9773; **(B) **multiple AZD9773 doses**.

**Figure 3:**
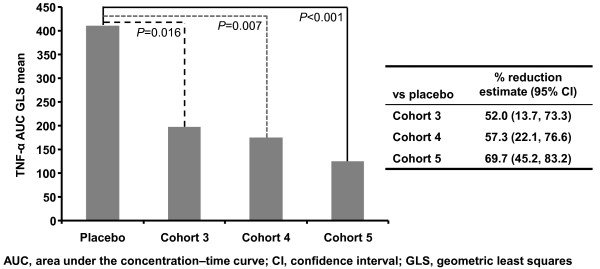
**Exploratory analysis of TNF-α AUC over treatment: multiple AZD9773 dose cohorts vs placebo**.

Median baseline TNF-α serum concentrations were generally similar across cohorts (range 3.6 to 5.8 pg/mL), except for AZD9773 cohort 2 (9.8 pg/mL). Any result below the LoQ was assigned that concentration (that is, 1.3 pg/mL). There was no discernible effect on total TNF-α concentrations in AZD9773 cohort 1, while in cohort 2, TNF-α concentrations decreased close to the LoQ within two hours (Figure 2A). In AZD9773 cohorts 3 to 5, significant TNF-α reductions observed within two hours post-infusion were maintained throughout treatment (Figure 2B). In concordance with AZD9773 exposure, TNF-α inhibition decreased when dosing was stopped (Figure 2B). In an exploratory analysis of TNF-α AUC over treatment, significant reductions in TNF-α concentrations were observed with maintenance AZD9773 treatment across all three multiple-dose cohorts compared with placebo (Figure 3).

#### IL-6 concentrations

Median IL-6 serum concentrations decreased in all AZD9773 cohorts (from baseline to 48 hours: 465 to 99 pg/mL in cohort 1; 189 to 85 pg/mL in cohort 2; 215 to 163 pg/mL in cohort 3; 1403 to 165 pg/mL in cohort 4; and 336 to 90 pg/mL in cohort 5). In the placebo group, the median decreased from 454 to 68 pg/mL. There were no obvious differences between individual AZD9773 cohorts and placebo.

#### Procalcitonin levels

Median baseline procalcitonin levels were 28.5, 4.8, 4.0, 16.5 and 15.1 ng/mL in AZD9773 cohorts 1 to 5, respectively, and 14.7 ng/mL in the placebo patients. Procalcitonin values at baseline were above the reference range (that is, > 0.5 ng/mL) in 60/65 patients (92.3%; Table 2). On Day 7, procalcitonin levels had decreased to 3.4, 0.4, 2.3, 0.6 and 1.5 ng/mL in AZD9773 cohorts and to 0.5 ng/mL in placebo patients.

### Safety of AZD9773

#### Adverse events

Most patients experienced at least one TEAE; the most common TEAEs overall were anemia, agitation and constipation (all *n *= 13, 18.6%; Table 5). The majority of TEAEs were mild or moderate in intensity and were not considered by the investigators to be related to study drug. Investigator-assessed drug-related TEAEs occurred in 0, 2 (22.2%), 2 (16.7%), 3 (30.0%) and 2 (25.0%) patients in AZD9773 cohorts 1 to 5, respectively, and in 10 patients (43.5%) who received placebo. In total, four patients discontinued treatment due to TEAEs (one AZD9773 patient each with cardiac arrest, sepsis and cerebral infarction, and atrial fibrillation in a placebo-treated patient). There was no overall difference in the type or severity of TEAEs or serious AEs between treatment cohorts (Tables 5 and 6).

**Table 5:** Most common treatment-emergent adverse events, irrespective of relationship to study drug (safety population)*

	AZD9773 cohort 1 (50 U/kg) (*n *= 8)	AZD9773 cohort 2 (250 U/kg) (*n *= 9)	AZD9773 cohort 3 (250/50 U/kg) (*n *= 12)	AZD9773 cohort 4 (500/100 U/kg) (*n *= 10)	AZD9773 cohort 5 (750/250 U/kg) (*n *= 8)	Placebo (*n *= 23)
Patients with any TEAE	8 (100.0)	9 (100.0)	12 (100.0)	8 (80.0)	7 (87.5)	23 (100.0)

Anemia	1 (12.5)	3 (33.3)	2 (16.7)	1 (10.0)	2 (25.0)	4 (17.4)
Agitation	2 (25.0)	2 (22.2)	1 (8.3)	3 (30.0)	1 (12.5)	4 (17.4)
Constipation	2 (25.0)	2 (22.2)	2 (16.7)	1 (10.0)	0	6 (26.1)
Generalized edema	0	1 (11.1)	3 (25.0)	2 (20.0)	3 (37.5)	3 (13.0)
Hypoglycemia	1 (12.5)	1 (11.1)	1 (8.3)	4 (40.0)	3 (37.5)	2 (8.7)
Hypokalemia	2 (25.0)	1 (11.1)	0	2 (20.0)	0	7 (30.4)
Hypernatremia	1 (12.5)	1 (11.1)	0	3 (30.0)	2 (25.0)	2 (8.7)
Hypophosphatemia	2 (25.0)	1 (11.1)	0	2 (20.0)	1 (12.5)	2 (8.7)
Diarrhea	2 (25.0)	0	0	1 (10.0)	1 (12.5)	4 (17.4)
Edema peripheral	2 (25.0)	0	0	0	1 (12.5)	4 (17.4)
Insomnia	1 (12.5)	2 (22.2)	0	1 (10.0)	0	2 (8.7)
Sepsis	1 (12.5)	0	3 (25.0)	0	0	1 (4.3)
Wheezing	1 (12.5)	2 (22.2)	0	1 (10.0)	0	1 (4.3)
Bradycardia	0	0	0	2 (20.0)	0	3 (13.0)
Feeding disorder	1 (12.5)	2 (22.2)	0	0	0	1 (4.3)
Decubitus ulcer	1 (12.5)	0	0	2 (20.0)	1 (12.5)	0
Hypertension	0	1 (11.1)	0	2 (20.0)	1 (12.5)	0
Scleral hemorrhage	2 (25.0)	0	0	0	0	0

*Data arranged in order of overall frequency (≥ 20% in any cohort)

**Table 6:** Serious treatment-emergent adverse events, irrespective of relationship to study drug (safety population)*

	AZD9773 cohort 1 (50 U/kg) (*n *= 8)	AZD9773 cohort 2 (250 U/kg) (*n *= 9)	AZD9773 cohort 3 (250/50 U/kg) (*n *= 12)	AZD9773 cohort 4 (500/100 U/kg) (*n *= 10)	AZD9773 cohort 5 (750/250 U/kg) (*n *= 8)	Placebo (*n *= 23)
Patients with any serious TEAE	3 (37.5)	6 (66.7)	7 (58.3)	3 (30.0)	4 (50.0)	13 (56.5)

Sepsis	1 (12.5)	0	3 (25.0)	0	0	1 (4.3)
Pneumonia	1 (12.5)	1 (11.1)	0	0	0	2 (8.7)
Septic shock	0	1 (11.1)	0	0	1 (12.5)	2 (8.7)
Respiratory failure	0	0	0	2 (20.0)	0	2 (8.7)
Cardiac arrest	1 (12.5)	1 (11.1)	0	0	0	1 (4.3)
Pulmonary embolism	0	1 (11.1)	0	0	1 (12.5)	1 (4.3)
Deep vein thrombosis	0	0	1 (8.3)	1 (10.0)	0	1 (4.3)
Hemorrhagic shock	0	0	1 (8.3)	0	0	1 (4.3)
Empyema	0	0	1 (8.3)	0	0	0
Endocarditis	0	1 (11.1)	0	0	0	0
Cellulitis	0	0	0	0	0	1 (4.3)
Acute myocardial infarction	0	0	0	1 (10.0)	0	0
Cardiac failure	0	0	1 (8.3)	0	0	0
Supraventricular tachycardia	0	0	0	1 (10.0)	0	0
Atrial fibrillation	0	0	0	0	0	1 (4.3)
Pneumonia aspiration	0	0	0	1 (10.0)	0	0
Hypoxia	0	0	0	0	0	1 (4.3)
Shock	0	0	1 (8.3)	0	0	0
Peripheral ischemia	0	0	0	0	0	1 (4.3)
Enterocutaneous fistula	0	0	0	0	1 (12.5)	0
Intestinal ischemia	0	0	0	0	1 (12.5)	0
Intestinal perforation	0	0	0	0	0	1 (4.3)
Medical device complication	0	1 (11.1)	0	0	0	0
Acidosis	0	1 (11.1)	0	0	0	0
Malignant lung neoplasm	0	1 (11.1)	0	0	0	0
Cerebral infarction	0	0	1 (8.3)	0	0	0
Intracranial hemorrhage	0	0	0	0	0	1 (4.3)
Toxic epidermal necrolysis	1 (12.5)	0	0	0	0	0
Muscle hemorrhage	0	0	0	0	0	1 (4.3)

*Data arranged in order of overall frequency

Hypoglycemia, generalized edema and hypernatremia occurred more frequently in some AZD9773 cohorts compared with other AZD9773 cohorts and placebo (Table 5).

#### Treatment-emergent infections

Nineteen of 47 patients (40.4%) who received AZD9773 experienced 31 treatment-emergent infections, while 10/23 (43.5%) who received placebo experienced 13 treatment-emergent infections. Of these 44 events, 10 had a fatal outcome (7 and 3 in the AZD9773 and placebo cohorts, respectively), 16 resolved (10 and 6 in the AZD9773 and placebo cohorts, respectively) and 18 were ongoing at the end of the study period (14 and 4 in the AZD9773 and placebo cohorts, respectively).

#### Laboratory data

Assessment of laboratory data showed changes within individual patients for a number of parameters. However, there was no clear evidence that the pattern of laboratory data differed between the AZD9773 cohorts and placebo patients and there was a general trend towards an improvement over time. Furthermore, there was no apparent effect of increased exposure to AZD9773 on laboratory parameters.

Total human anti-sheep antibody (HASA) and HASA IgG levels were undetectable at baseline. Following AZD9773 treatment, total HASA levels were positive in one patient in cohort 3 and five patients in cohort 4, and undetected in the remainder of the patients. HASA IgG was positive following AZD9773 treatment in two patients in cohort 3, three patients in cohort 4 and two patients in cohort 5.

#### Electrocardiogram assessment

Small fluctuations in ECG evaluations around baseline values were observed during treatment for all parameters within all groups, although there were no notable differences or obvious trends. AZD9773 had no discernible effect on QTc interval.

#### Mortality

The overall 28-day mortality was 27.7% (*n *= 13/47) across the AZD9773 cohorts and 26.1% (*n *= 6/23) in placebo patients (Table 7). The most common causes of death were sepsis and septic shock (*n *= 4 for each), and pneumonia and respiratory failure (*n *= 2 for each). No deaths were considered by the investigator to be related to study drug.

**Table 7:** 28-day mortality (safety population)

	AZD9773 cohort 1 (50 U/kg) (*n *= 8)	AZD9773 cohort 2 (250 U/kg) (*n *= 9)	AZD9773 cohort 3 (250/50 U/kg) (*n *= 12)	AZD9773 cohort 4 (500/100 U/kg) (*n *= 10)	AZD9773 cohort 5 (750/250 U/kg) (*n *= 8)	Placebo (*n *= 23)
Alive, n (%)	6 (75.0)	5 (55.6)	7 (58.3)	9 (90.0)	7 (87.5)	16 (69.6)
Dead, n (%)	2 (25.0)	4 (44.4)	5 (41.7)	1 (10.0)	1 (12.5)	6 (26.1)
Lost to follow-up, n (%)	0	0	0	0	0	1 (4.3)

### Sepsis care

The median number of shock-free days on Day 7 was 4.0 in the placebo group and ranged from 2.0 to 4.5 in the AZD9773 cohorts; equivalent values on Day 14 were 11.0 in the placebo group and 7.5 to 11.5 in the AZD9773 cohorts (see Additional file 4). The median number of ventilator-free days was 19.0 in the placebo group and ranged from 8.0 to 22.5 in the AZD9773 cohorts (see Additional file 4).

#### Concomitant medication

Concomitant drotrecogin alfa was received by 16 patients (22.9%) during the study, including 10/47 (21.3%) AZD9773 patients and 6/23 (26.1%) placebo patients. For patients receiving drotrecogin alfa, the median APACHE II score was 31 (range 13 to 41) in the AZD9773 cohorts and 27 (range 21 to 37) in the placebo group.

Forty-two patients (60%) received concomitant corticosteroids during treatment, predominantly, but not exclusively, early in the septic period; this comprised 28/47 (59.6%) AZD9773 patients and 14/23 (60.9%) placebo patients. There were seven deaths due to infection in AZD9773-treated patients and all received concomitant corticosteroids. There were three deaths due to infection in the placebo-treated patients; one received concomitant corticosteroids.

#### Sequential Organ Failure Assessment scores

In general, median SOFA scores decreased during the treatment period (see Additional file 5). The total organ failure resolution rate was 57.4% (27/47) in the AZD9773 cohorts and 60.9% (14/23) in the placebo group.

## Discussion

This double-blind, randomized Phase IIa study demonstrated that at the dose regimens and infusion rates studied, AZD9773 was well tolerated with an acceptable safety profile. Although earlier studies of TNF-α suppression have been associated with only modest clinical benefit, AZD9773 has a number of potential advantages over previously evaluated agents [9]. As a polyclonal Fab fragment, it binds to multiple domains of TNF-α and is likely to exhibit improved tissue penetration [9-12]. A previous Phase II efficacy study with CytoFab demonstrated an increased number of ventilator- and ICU-free days, with a trend towards improved mortality, compared with placebo in patients with severe sepsis [13]. Informal comparison between the safety and PK/PD data reported here and previously reported CytoFab data [13] suggest that changes to the manufacturing process have not adversely impacted the safety or tolerability profile of the drug.

Most patients in both the AZD9773 cohorts and placebo group completed the treatment period (63/70; 90.0%). In general, the AZD9773 cohorts and placebo patients were comparable for safety and tolerability, and there was no apparent increase in the incidence or severity of AEs or changes in laboratory parameters with increasing exposure to AZD9773. Although progression of sepsis and its treatment confounded detailed analysis, there were no apparent patterns in TEAE or laboratory data, or consistent relationship to administration of AZD9773, to support an association between AZD9773 treatment and a specific TEAE. The incidence of TEAEs considered at the time of the event as being related to study drug by the reporting investigator was lower in patients who received AZD9773 than in those who received placebo. The 28-day mortality rate was similar between placebo and AZD9773-treated patients and, although the number of patients in each cohort was small, was in keeping with expected mortality rates in this patient population.

Across the doses studied, AZD9773 exposure increased in an approximately dose-proportional manner. Large inter-patient variability in exposure was observed, although a population PK/PD analysis demonstrated a potential relationship with creatinine clearance (CrCL) [23]. Patients with lower CrCL had higher AZD9773 exposure; however, the increase was not expected to exceed a 10-fold range within this patient population. Patients with renal dysfunction were not excluded from this study and, although low CrCL rates were observed, there was no increase in adverse events in these patients due to exposure. There was no additional impact on AZD9773 exposure in patients on dialysis compared with other study patients. Although both total and specific AZD9773 Fabs were detected in dialysate samples, there was no evidence of reduced exposure to AZD9773 in serum from patients with renal dysfunction.

Approximately 15% of AZD9773 was excreted in urine. Comparison of renal clearance to total serum clearance suggests that between 25% and 33% of drug clearance is renal. This indicates that renal elimination plays an important role in the clearance of AZD9773, although most is likely to be cleared through the reticuloendothelial system. Current studies do not preclude administration of AZD9773 to patients with renal impairment.

The terminal t_1/2 _of AZD9773 was approximately 20 hours and was multi-phasic, with most inhibitory activity occurring within the first phase, resulting in an immediate suppression of TNF-α levels. In AZD9773 cohorts 3 to 5, TNF-α detection declined significantly within two hours post-infusion, and the apparent TNF-α suppression was maintained throughout treatment (Figure 2B). While it has not been possible to formally demonstrate a loss of TNF-α bioactivity following AZD9773 dosing (due to assay feasibility issues), it is highly likely that TNF-α bioactivity is correspondingly diminished. AZD9773 is a polyclonal TNF-α-neutralizing agent, and characterization of the AZD9773:TNF-α interaction reveals that multiple AZD9773 Fabs bind per TNF-α monomer [24]. Indeed, AZD9773 competes with various commercially available TNF-α detection Ab systems and novel phage-Ab anti-TNF binding antibody fragments (data not shown), making detection of TNF-α in the presence of AZD9773 problematic. However, the typical molar ratios of AZD9773:TNF-α present in the serum of patients in this study make it highly likely that TNF-α bioactivity will be completely neutralized.

The relatively short t_1/2 _of AZD9773 resulted in an immediate increase in TNF-α levels following cessation of dosing, with the rapid 80 to 90% decline in serum concentration observed within the first 12 hours facilitating the subsequent normalization of TNF-α levels. Although there was no apparent impact of AZD9773 on IL-6 and procalcitonin levels compared with placebo, these parameters will continue to be assessed in larger studies that are specifically designed to assess efficacy outcomes.

A previously reported population PK/PD analysis demonstrated a proportional relationship between TNF-α suppression and AZD9773 exposure [23]. The PK profiles and the concentration-effect relationships with TNF-α were comparable between AZD9773 and CytoFab [13]. The population PK/PD model was used to simulate potential doses for use in future clinical studies, from which a 750 U/kg loading dose followed by 250 U/kg maintenance doses was deemed unlikely to provide any additional overall improvement in TNF-α suppression compared with 500 U/kg loading and 100 U/kg maintenance doses, or 250 U/kg loading and 50 U/kg maintenance doses. These latter two doses are therefore being employed in the ongoing Phase IIb studies (ClinicalTrials.gov identifiers: NCT01145560 and NCT01144624).

As an exogenous protein, AZD9773 might be expected to generate an immunological reaction in some patients. However, this study had limited ability to fully characterize any potential reaction for a variety of reasons: the small number of patients in each cohort; concomitant medications received; the disease under study; and the relatively short duration of follow-up.

The incidence of recipient antibody formation reported with the use of biological therapies varies widely in the literature, ranging from 0 to 100% depending on the type of product and patient population [25]. Here, HASAs were not expected to reduce AZD9773 efficacy, given the time required for their formation relative to the short duration of treatment. A small number of patients developed HASAs in this study (6/47; 12.8%), the incidence of which was not dose related and was lower than that reported with CytoFab (11/27; 40.7%) [13]. Data from similar products, such as crotalidae polyvalent immune Fab (CroFab^®^, BTG International Inc., London, UK) [26] and digoxin immune Fab (Digibind^®^, BTG International Inc., London, UK) [27], also suggest that HASAs are unlikely to pose a significant safety risk, given the short duration of treatment proposed for AZD9773.

An important limitation in this study was the small patient sample size, as the study population was not sufficiently large to detect clinical benefit.

## Conclusions

In conclusion, the data support further assessment of this ovine-derived, anti-TNF-α antibody fragment in patients with severe sepsis. Larger, randomized, global Phase IIb clinical trials are ongoing to further characterize the safety and efficacy of AZD9773 in this patient population.

## Key messages

■ TNF-α, an early mediator in the systemic inflammatory response to infection, is a therapeutic target in sepsis. AZD9773 is an ovine-derived, polyclonal, anti-human TNF-α Fab preparation in development for the treatment of patients with severe sepsis and septic shock.

■ This double-blind, randomized Phase IIa study showed that most adverse events during AZD9773 treatment were of mild-to-moderate intensity and considered by the investigator as unrelated to study treatment.

■ AZD9773 pharmacokinetic data demonstrated an approximately proportional increase in concentration with increasing dose and a terminal half-life of 20 hours.

■ Serum TNF-α concentrations decreased to near-undetectable levels within two hours of commencing AZD9773 infusion.

■ The safety, pharmacokinetic and pharmacodynamic data support the continued evaluation of AZD9773 in larger Phase IIb/III studies.

## Abbreviations

AEs: adverse effects; APACHE II: Acute Physiology and Chronic Health Evaluation II; AUC_0-12_: area under the serum concentration-time curve from 0 to 12 hours; CI: confidence interval; C_inf_: maximum (end of infusion) serum concentration; CL: total apparent drug clearance; COPD: chronic obstructive pulmonary disease; CrCL: creatinine clearance; CV: coefficient of variation; ECG: electrocardiogram; EOI: end of infusion; Fab: fragment antigen binding; GLS: geometric least squares; HASA: human anti-sheep antibody; ICU: intensive care unit; IDMC: Independent Data Monitoring Committee; IL: interleukin; LoQ: limit of quantification; PD: pharmacodynamics; PK: pharmacokinetics; rh: recombinant human; SD: standard deviation; SOFA: Sequential Organ Failure Assessment; t_1/2_: terminal half-life; TEAE: treatment-emergent adverse event; t_max_: time to reach C_inf; _TNF-α: tumor necrosis factor-α; V_SS_: apparent volume of distribution at steady state

## Competing interests

PEM, BZ and ACB have no competing interests to declare. GRB has received research grants from AstraZeneca. SS is a former employee of AstraZeneca and holds stock in the company; he is currently an employee of Agennix Inc. XH, SD and TE are current employees of AstraZeneca.

## Authors' contributions

PEM, BZ and ACB served as investigators on this trial, enrolling patients. PEM, BZ, ACB and GRB contributed to data interpretation, and reviewed and provided their comments on this manuscript. GRB, SS, SD and TE were involved in the design of the study. SS, SD and TE were involved in the execution of the study and contributed to the analysis, interpretation and reporting of the data. XH served as the study statistician. All authors read and approved the final manuscript.

## Supplementary Material

Additional file 1 **Study definitions**. A list of definitions for the inclusion criteria of the study. Click here for file

Additional file 2 **Table S1**. Organ dysfunction definitions. Table listing the definitions of organ dysfunction used in the study, to go with Additional file 1. Click here for file

Additional file 3 **Figure S1**. Timing of severe sepsis inclusion criteria. Diagram showing the timescales for the inclusion criteria, to go with Additional file 1. Click here for file

Additional file 4 **Shock- and ventilator-free days (safety population)**. Table showing the number of shock- and ventilator-free days in the safety population. Click here for file

Additional file 5 **Median (range) SOFA scores over time (safety population)**. Table showing the SOFA scores in the safety population over time. Click here for file
