# Supplementary material for: A placebo-controlled, double-blind, dose-escalation study to assess the safety, tolerability and pharmacokinetics/pharmacodynamics of single and multiple intravenous infusions of AZD9773 in patients with severe sepsis and septic shock
Source: Crit Care. 2012 Feb 17;16(1):R31. doi: 10.1186/cc11203 (PMC3396277; doi:10.1186/cc11203)
Supplement: Additional file 1 — Study definitions. A list of definitions for the inclusion criteria of the study. [file cc11203-S1.DOCX]

**Additional file 1: Study definitions**

1. Clinical evidence of infection requiring treatment with parenteral antibiotics as evidenced by one of the following (also see criterion 4):
   1. Perforated viscus
   2. White blood cells (WBC) and/or pathogens in a normally sterile body fluid
   3. Radiographic evidence of pneumonia associated with the production of purulent sputum
   4. Signs of a local source of infection such as cellulitis
   5. A syndrome associated with a high risk of infection (eg, ascending cholangitis)
   6. Positive culture from blood or from another normally sterile body fluid prior to study drug administration
2. Patients must meet at least three of the following four SIRS criteria (also see criterion 4) – these criteria do not have to be met simultaneously; the actual values and the date and time criteria were met will be collected:
   1. Core temperature of >38°C or <36°C measured via tympanic, oral, rectal (preferred), or thermistor method
   2. Heart rate of >90 beats/min
   3. Respiratory rate of >20 breaths/min related to septic event, or partial pressure of arterial carbon dioxide (PaCO_2_) <32 mmHg related to septic event, or requiring mechanical ventilation related to septic event
   4. Total WBC absolute count >12000 cells/mm^3^ or <4000 cells/mm^3^. In the presence of granulocyte stimulating factor, all three other vital sign criteria must be met
3. Patients must meet criteria for cardiovascular and/or respiratory dysfunction (also see criterion 4). Newly developed organ dysfunctions must be in the context of the acute septic process not explained by a chronic condition or by effects of concomitant therapy. Time of first organ failure will be recorded. Organ dysfunction definitions are provided in Table S1.
4. Sepsis (infection + SIRS criteria) must be present prior to cardiovascular and/or respiratory dysfunction. SIRS criteria and cardiovascular and/or respiratory dysfunction do not need to be present simultaneously, but SIRS criteria must have been met within the 24 hours preceding the initial cardiovascular and/or respiratory dysfunction. Study drug administration must occur within 36 hours after the cardiovascular and/or respiratory dysfunction resulting in severe sepsis. The timing of development of severe sepsis inclusion criteria is presented in Figure S1.
