# Supplementary material for: A placebo-controlled, double-blind, dose-escalation study to assess the safety, tolerability and pharmacokinetics/pharmacodynamics of single and multiple intravenous infusions of AZD9773 in patients with severe sepsis and septic shock
Source: Crit Care. 2012 Feb 17;16(1):R31. doi: 10.1186/cc11203 (PMC3396277; doi:10.1186/cc11203)
Supplement: Additional file 2 — Table S1. Organ dysfunction definitions. Table listing the definitions of organ dysfunction used in the study, to go with Additional file 1. [file cc11203-S2.DOCX]

**Additional file 2: Table S1. Organ dysfunction definitions**

| **System/organ** | **Definition** |
| --- | --- |
| Cardiovascular system | Hypotension, as defined by a systolic blood pressure <90 mmHg, or a mean arterial blood pressure ≤65 mmHg (if measured via an arterial line) for at least 1 hour in the face of adequate filling pressures when measured or unresponsive to saline infusion (20 mL/kg), or requiring pressor support to maintain blood pressure greater than above limits. If less than 20 mL/kg of fluid has been given before initiating pressors, documentation of filling pressures is required |
| Pulmonary dysfunction | If pulmonary dysfunction is the only organ dysfunction for study entry, the patient must require mechanical ventilation related to the septic process and have a PaO_2_/FiO_2_ ≤300, if lung is not the primary site of infection. If lung is the primary point of infection, PaO_2_/FiO_2_ must be <200. Acute respiratory distress syndrome will be noted on the eCRF if the patient’s condition fulfils the following criteria: PaO_2_/FiO_2_ ≤200 with acute bilateral diffuse infiltrates on chest X-ray compatible with pulmonary oedema (infiltrates may be patchy, diffuse, homogenous or asymmetric, but must not be explained by lung masses, segmental or lobar atelectasis or pleural effusion) and pulmonary artery wedge pressure ≤18 mmHg (if measured) |

FiO_2_: fraction of inspired oxygen; PaO_2_: partial pressure of arterial oxygen
