# Supplementary material for: A placebo-controlled, double-blind, dose-escalation study to assess the safety, tolerability and pharmacokinetics/pharmacodynamics of single and multiple intravenous infusions of AZD9773 in patients with severe sepsis and septic shock
Source: Crit Care. 2012 Feb 17;16(1):R31. doi: 10.1186/cc11203 (PMC3396277; doi:10.1186/cc11203)
Supplement: Additional file 3 — Figure S1. Timing of severe sepsis inclusion criteria. Diagram showing the timescales for the inclusion criteria, to go with Additional file 1. [file cc11203-S3.DOCX]

**Additional file 3: Figure S1. Timing of severe sepsis inclusion criteria**

^a^Sepsis (infection + SIRS criteria) must be present prior to organ failure (cardiovascular and/or respiratory dysfunction); ^b^SIRS criteria and organ failure (cardiovascular and/or respiratory dysfunction) do not need to be present simultaneously but SIRS criteria must have been met within the 24 hours preceding the initial organ failure; ^c^Study drug administration must occur within 36 hours after organ failure (cardiovascular and/or respiratory dysfunction) resulting in severe sepsis
