# Supplementary material for: A placebo-controlled, double-blind, dose-escalation study to assess the safety, tolerability and pharmacokinetics/pharmacodynamics of single and multiple intravenous infusions of AZD9773 in patients with severe sepsis and septic shock
Source: Crit Care. 2012 Feb 17;16(1):R31. doi: 10.1186/cc11203 (PMC3396277; doi:10.1186/cc11203)
Supplement: Additional file 4 — Shock- and ventilator-free days (safety population). Table showing the number of shock- and ventilator-free days in the safety population. [file cc11203-S4.DOCX]

**Additional file 4: Shock- and ventilator-free days (safety population)**

|  | **AZD9773 cohort 1**  **(50 U/kg)** | **AZD9773 cohort 2**  **(250 U/kg)** | **AZD9773 cohort 3**  **(250/50 U/kg)** | **AZD9773 cohort 4**  **(500/100 U/kg)** | **AZD9773 cohort 5**  **(750/250 U/kg)** | **Placebo** |
| --- | --- | --- | --- | --- | --- | --- |
| Days alive and shock-free, up to and including day 14 | | | | | | |
|  | *(n=8)* | *(n=9)* | *(n=12)* | *(n=10)* | *(n=8)* | *(n=23)* |
| Mean ± SD | 10.6 ± 3.5 | 8.0 ± 6.3 | 6.5 ± 6.1 | 8.1 ± 4.6 | 9.3 ± 4.7 | 9.6 ± 4.3 |
| Median (range) | 11.0 (4–14) | 11.0 (0–14) | 7.5 (0–14) | 9.5 (0–14) | 11.5 (0–14) | 11.0 (0–14) |
| Days alive and shock-free, up to and including day 7 | | | | | | |
|  | *(n=8)* | *(n=9)* | *(n=12)* | *(n=10)* | *(n=8)* | *(n=23)* |
| Mean ± SD | 4.6 ± 2.4 | 3.3 ± 3.1 | 2.9 ± 3.2 | 2.8 ± 2.4 | 3.5 ± 2.8 | 3.7 ± 2.4 |
| Median (range) | 4.0 (0–7) | 4.0 (0–7) | 2.0 (0–7) | 2.5 (0–7) | 4.5 (0–7) | 4.0 (0–7) |
| Ventilator-free days | | | | | | |
|  | *(n=8)* | *(n=9)* | *(n=12)* | *(n=10)* | *(n=8)* | *(n=23)* |
| Mean ± SD | 16.5 ± 10.8 | 10.6 ± 10.8 | 11.3 ± 12.1 | 14.6 ± 10.4 | 13.9 ± 11.7 | 14.9 ± 11.2 |
| Median (range) | 22.5 (0–26) | 13.0 (0–26) | 8.0 (0–28) | 19.0 (0–26) | 19.5 (0–26) | 19.0 (0–28) |

*150% of the target volume [<6 mL/kg] recommended in the Surviving Sepsis Campaign Guidelines [2]
