# Supplementary material for: A placebo-controlled, double-blind, dose-escalation study to assess the safety, tolerability and pharmacokinetics/pharmacodynamics of single and multiple intravenous infusions of AZD9773 in patients with severe sepsis and septic shock
Source: Crit Care. 2012 Feb 17;16(1):R31. doi: 10.1186/cc11203 (PMC3396277; doi:10.1186/cc11203)
Supplement: Additional file 5 — Median (range) SOFA scores over time (safety population). Table showing the SOFA scores in the safety population over time. [file cc11203-S5.DOCX]

**Additional file 5: Median (range) SOFA scores over time (safety population)**

|  | **AZD9773 cohort 1**  **(50 U/kg)** | **AZD9773 cohort 2**  **(250 U/kg)** | **AZD9773 cohort 3**  **(250/50 U/kg)** | **AZD9773 cohort 4**  **(500/100 U/kg)** | **AZD9773 cohort 5**  **(750/250 U/kg)** | **Placebo** |
| --- | --- | --- | --- | --- | --- | --- |
| Baseline | *(n=7)* | *(n=8)* | *(n=7)* | *(n=8)* | *(n=6)* | *(n=20)* |
|  | 11.0 (6–20) | 10.5 (6–16) | 10.0 (6–16) | 12.5 (7–14) | 11.5 (5–14) | 11.0 (5–16) |
| Day 1 | *(n=7)* | *(n=6)* | *(n=8)* | *(n=10)* | *(n=5)* | *(n=16)* |
|  | 10.0 (6–19) | 9.5 (6–16) | 10.5 (5–18) | 11.5 (3–15) | 9.0 (7–16) | 12.0 (3–16) |
| Day 3 | *(n=5)* | *(n=4)* | *(n=8)* | *(n=7)* | *(n=4)* | *(n=16)* |
|  | 7.0 (4–10) | 10.5 (4–12) | 9.0 (2–19) | 9.0 (6–15) | 7.0 (0–8) | 8.0 (2–18) |
| Day 5 | *(n=6)* | *(n=2)* | *(n=5)* | *(n=7)* | *(n=6)* | *(n=11)* |
|  | 6.0 (4–10) | 8.5 (5–12) | 7.0 (2–14) | 8.0 (5–14) | 4.5 (0–10) | 6.0 (3–15) |
| Day 6 | *(n=2)* | *(n=3)* | *(n=5)* | *(n=6)* | *(n=3)* | *(n=9)* |
|  | 6.5 (6–7) | 9.0 (5–9) | 6.0 (2–14) | 9.0 (5–14) | 1.0 (0–5) | 6.0 (3–18) |
| Total organ failure resolution, n/N (%)* | 4/8 (50.0) | 4/9 (44.4) | 6/12 (50.0) | 8/10 (80.0) | 5/8 (62.5) | 14/23 (60.9) |

*N, overall number of patients in cohort; n, number of patients with total resolution
